# The Pharmacokinetics of BPA: Similarities in Human and Animal Metabolism Suggest Higher Exposure than Thought

**DOI:** 10.1289/ehp.119-a177a

**Published:** 2011-04

**Authors:** Julia R. Barrett

**Affiliations:** **Julia R. Barrett**, MS, ELS, a Madison, WI–based science writer and editor, has written for *EHP* since 1996. She is a member of the National Association of Science Writers and the Board of Editors in the Life Sciences

Bisphenol A (BPA) has been shown to cause adverse health effects in animals, but attempts to extrapolate human health effects from this evidence are impeded by unanswered questions about routes and levels of exposure, metabolism, and whether animal models are appropriate proxies for humans. New findings show the kinetics of BPA metabolism are very similar in humans, monkeys, and mice and also suggest greater human exposure than previously estimated **[*EHP*** **119(4):422–430; Taylor et al.]**.

BPA in food and beverage packaging likely underlies most human oral exposure, and dermal and inhalation exposure may occur from other sources. BPA has been assumed to undergo rapid metabolism (conjugation) and clearance from the body. However, recent human biomonitoring data showed serum concentrations of unconjugated BPA, the bioactive form, at levels much higher than predicted given earlier assumptions about the amount of BPA ingested by humans and its expected rate of clearance.

The authors of the current study studied clearance of radiolabeled unconjugated BPA in rhesus macaques and CD-1 mice, then compared the results with those from a previous oral dosing study in women. In the first experiment, female monkeys received deuterated BPA (dBPA) at 400 μg per kg body weight once a day for a week. Blood samples were collected prior to dosing and several times on days 1 and 7. The second experiment involved an oral dose of 400 μg ^3^H-BPA per kg body weight to female CD-1 mice and measurements of the unconjugated compound in serum over the next 24 hours. A second group of mice received a single dose of varying amounts of ^3^H-BPA, with unconjugated serum levels measured 24 hours later, and a third group received a single dose of BPA at 100,000 μg/kg, with serum assessed for unconjugated BPA several times over the next 24 hours.

Unconjugated dBPA concentrations in monkeys averaged 0.5 ng/mL over 24 hours and peaked at 3.94 ng/mL 1 hour after treatment. These values are comparable to medians of 0.3–4.0 ng/mL reported in human biomonitoring studies. The amount of BPA needed to achieve the serum concentrations in monkeys far exceeded the 2007 U.S. Food and Drug Administration human exposure estimate of 0.16 μg/kg/day as well as the U.S. Environmental Protection Agency’s daily intake dose of 50 μg/kg. Results from the mouse experiments showed a linear relationship between BPA dose and unconjugated BPA in serum, with the kinetics of metabolism remarkably similar to those observed in monkeys and humans.

If the reported plasma BPA concentrations in humans are accurate, the results suggest human exposure is currently underestimated and that there may be significant sources of exposure though non-oral routes. Additionally, they support CD-1 mouse studies as being relevant for estimating serum levels of unconjugated BPA in humans.

